# Carvacrol inhibits the progression of oral submucous fibrosis via downregulation of PVT1/miR‐20a‐5p‐mediated pyroptosis

**DOI:** 10.1111/jcmm.70112

**Published:** 2024-09-25

**Authors:** Cheng‐Chia Yu, Pei‐Ling Hsieh, Shih‐Chi Chao, Yi‐Wen Liao, Chuan‐Hang Yu, Pin Ju Chueh, Chih‐Yu Peng, Shiuan‐Shinn Lee

**Affiliations:** ^1^ Institute of Oral Sciences, Chung Shan Medical University Taichung Taiwan; ^2^ Department of Dentistry Chung Shan Medical University Hospital Taichung Taiwan; ^3^ School of Dentistry, Chung Shan Medical University Taichung Taiwan; ^4^ Oral Medicine Research Center Chung Shan Medical University Taichung Taiwan; ^5^ Department of Anatomy, School of Medicine China Medical University Taichung Taiwan; ^6^ Department of Medical Research Chung Shan Medical University Hospital Taichung Taiwan; ^7^ Institute of Biomedical Sciences, National Chung Hsing University Taichung Taiwan; ^8^ Department of Post‐Baccalaureate Medicine College of Medicine, National Chung Hsing University Taichung Taiwan; ^9^ Department of Public Health College of health care and management, Chung Shan Medical University Taichung Taiwan

**Keywords:** carvacrol, microRNA‐20a, myofibroblast, oral submucous fibrosis, PVT1

## Abstract

Oral submucous fibrosis (OSF) is a precancerous condition in the oral cavity, which is closely related to the myofibroblast conversion of buccal mucosal fibroblasts (BMFs) after chronic consumption of areca nut. Emerging evidence suggests pyroptosis, a form of programmed cell death that is mediated by inflammasome, is implicated in persistent myofibroblast activation and fibrosis. Besides, numerous studies have demonstrated the effects of non‐coding RNAs on pyroptosis and myofibroblast activities. Herein, we aimed to target key long non‐coding RNA PVT1 with natural compound, carvacrol, to alleviate pyroptosis and myofibroblast activation in OSF. We first identified PVT1 was downregulated in the carvacrol‐treated fBMFs and then demonstrated that myofibroblast features and expression of pyroptosis makers were all reduced in response to carvacrol treatment. Subsequently, we analysed the expression of PVT1 and found that PVT1 was aberrantly upregulated in OSF specimens and positively correlated with several fibrosis markers. After revealing the suppressive effects of carvacrol on myofibroblast characterisitcs and pyroptosis were mediated by repression of PVT1, we then explored the potential mechanisms. Our data showed that PVT1 may serve as a sponge of microRNA(miR)‐20a to mitigate the myofibroblast activation and pyroptosis. Altogether, these findings indicated that the anti‐fibrosis effects of carvacrol merit consideration and may be due to the attenuation of pyroptosis and myofibroblast activation by targeting the PVT1/miR‐20a axis.

## 1 INTRODUCTION

Pathological fibrosis is a condition due to an excessive accumulation of connective tissue in the extracellular matrix (ECM) following a reparative response to injury or damage, which may eventually result in organ malfunction or scarring. When this process occurs in the oral cavity, it is known as oral submucous fibrosis (OSF). OSF has been recognized as one of the oral potentially malignant disorders that are associated with areca nut chewing.^1^ It has been demonstrated that arecoline, the main alkaloid in areca nut, induced myofibroblast transdifferentiation from buccal mucosal fibroblasts (BMFs) as it elicited the formation of myofibroblast marker α‐smooth muscle actin (α‐SMA)‐positive stress fibres in BMFs and elevated myofibroblast activities.^2^ As the primary ECM‐secreting cells during wound healing and pathological fibrosis, myofibroblasts are often considered as attractive cell targets in regard to the development of treatment. Among various factors that regulate myofibroblast activation, pyroptosis has drawn increasing attention in recent years.

Pyroptosis is a form of programmed cell death (PCD) initiated by inflammasomes in response to pathogen infection, cellular stress or immune cell‐mediated attack. It is distinct from other types of PCD, such as apoptosis, necroptosis, autophagy, ferroptosis and cuproptosis in morphological features. The NOD‐like receptor family, pyrin domain‐containing‐3 (NLRP3) inflammasome has been known as the molecule most closely related to the initiation of pyroptosis as it can recruit procaspase‐1 via adapter molecule apoptosis‐associated speck‐like protein (ASC), leading to activation of caspase‐1 and the cleavage of gasdermin D (GSDMD).^3^ Cleavage of GSDMD has been recently discovered to be an executor of pyroptosis, which was associated with pore formation, plasma membrane rupture, cell lysis and interleukin(IL)‐1β/18 release.^4^, ^5^ Multiple lines of evidence have suggested that pyroptosis was implicated in the development of fibrosis. It has been shown that activation of NLRP3 inflammasome in alveolar epithelial cells enhanced the myofibroblast differentiation of lung‐resident mesenchymal stem cells and promoted pulmonary fibrosis.^6^ Another study has shown that deficiency of GSDMD ameliorated the folic acid‐induced renal fibrosis and the GSDMD‐dependent neutrophil extracellular traps formation promoted macrophage‐to‐myofibroblast transition.^7^ Most importantly, one recent study has demonstrated that the arecoline‐induced migration and collagen synthesis in human oral mucosal myofibroblast was mediated by the activation of the NLRP3 inflammasome complex.^8^ Consequently, a better understanding of the regulatory mechanism of pyroptosis may aid in exploring a therapeutic strategy for mitigating the activation of myofibroblasts and fibrosis.

Among various factors that initiate or regulate pyroptosis and myofibroblast activation, non‐coding RNAs have become the focus of numerous research studies. Non‐coding RNAs constitute more than 90% of the RNAs, but most of them lack protein‐coding capacity and remain largely uninvestigated. They can be classified into numerous groups based on their size, such as microRNAs (miRs; approximately 21–23 nucleotides) or long non‐coding RNAs (lncRNAs; greater than 200 nucleotides in length). Emerging evidence has suggested that lncRNAs can function as competing endogenous RNAs (ceRNAs) to regulate the effect of miRs on various biological processes, including the development of OSF.^9^, ^10^ Moreover, several studies have demonstrated that the ceRNA network is an integral part of the regulation of myofibroblast transdifferentiation through pyroptosis. For instance, the lncRNA MIR22HG/miR‐9‐3p/IGF1 axis has been shown to be involved in liver fibrosis in nonalcoholic steatohepatitis by promoting pyroptosis.^11^ In diabetic cardiomyopathy, lncRNA Kcnq1ot1 has been found to act as a ceRNA to modulate the expression of caspase‐1 by sponging miR‐214‐3p and the subsequent GSDMD cleavage as well as secretion of IL‐1β in high glucose‐treated cardiac fibroblasts.^12^ Lately, silencing lncRNA plasmacytoma variant translocation 1 (PVT1) has been demonstrated to mitigate myocardial ischemia–reperfusion (I/R) damage via suppressing GSDMD‐mediated pyroptosis.^13^ PVT1 was found to be upregulated and used for predicting resistance to cisplatin in ovarian cancer patients.^14^ PVT1 also modulated the expression of caspase 6 and caspase 8 via sequestering miR‐16‐5p, thereby affecting pyroptosis in pancreatic adenocarcinoma.^15^ Another study has shown that PVT1 regulated the NLRP3‐mediated pyroptosis in septic acute kidney injury by inhibiting miR‐20a‐5p.^16^ Given that lncRNA PVT1 participated in the regulation of pyroptosis, myofibroblast activation^17^, ^18^, ^19^ and oral carcinogenesis,^20^, ^21^ we speculated that PVT1 might also contribute to the development of precancerous OSF.

Multiple phytochemical and nutraceutical compounds have been found to regulate the expression of non‐coding RNAs and may be beneficial to the treatment of cancer^17^ or fibrosis.^18^ In the present study, we analysed the effect of carvacrol, a monoterpene phenol rich in essential oils of various aromatic plants, on suppression of arecoline‐induced myofibroblast activation as it was reported to modulate pyroptosis,^19^ fibrosis^22^ and oral carcinogenesis.^23^, ^24^, ^25^ However, its effects on suppression of precancerous OSF have not been investigated. We identified that PVT1 was one of the differentially expressed lncRNAs in the carvacrol‐treated fibrotic buccal mucosal fibroblasts (fBMFs) derived from OSF tissues. We then explored the functional role of PVT1 in myofibroblast activation and the mechanism by which PVT1 affects pyroptosis and myofibroblast transdifferentiation.

## 2 MATERIALS AND METHODS

### 2.1 Tissue collection, primary culture and reagents

All procedures adhered to the protocol approved by the Institutional Review Board of Chung Shan Medical University Hospital (CSMUH No.: CS17137). Following the acquisition of patients' consent, healthy and OSF tissues obtained from surgery were promptly immersed in phosphate‐buffered saline (PBS) for primary culture or in liquid nitrogen for subsequent PVT1 expression quantification. Normal buccal mucosal fibroblasts (BMFs) and fBMFs were extracted from fresh healthy buccal mucosa and OSF tissues, respectively. The tissues were cut into small pieces (0.5–1.0 mm^2^) and incubated with 0.05% trypsin–EDTA at 37°C for 30 min. Post‐centrifugation at 1200 rpm, the tissue pellets were plated into a 10‐cm culture dish with growth medium and maintained at 37°C/5% CO₂. After 7–14 days of incubation, spindle‐shaped cells that had migrated from the tissues were collected and routinely maintained in a growth medium composed of 90% Dulbecco's Modified Eagle Medium (DMEM), 10% fetal bovine serum (FBS), 100 U/mL penicillin and 100 μg/mL streptomycin. Cells from the third to the eighth passages were utilized in this study. Unless otherwise specified, all reagents were procured from Sigma (St. Louis, MO, USA).^26^

### 2.2 Cell proliferation and survival assay

BMF and fBMF cells were seeded into a 96‐well plate at a density of 1.0 × 10^4^ cells per well and incubated for 24 h. The medium was then replaced with a fresh culture medium containing varying concentrations of carvacrol (0, 2.5, 5 and 10 μM) for an additional 48 h of incubation. After this period, the proliferation rate and IC50 value were assessed using the MTT assay, following the manufacturer's instructions (Sigma‐Aldrich, St. Louis, MO, USA). Absorbance at 570 nm was measured with a microplate reader (Molecular Devices, San Jose, CA, USA).

### 2.3 Collagen gel contraction assay

Cells were embedded in a type I collagen gel solution (2 ng/mL) at a density of 2.0 × 10^5^ cells per well in a 24‐well plate. The cells and gel solution were gently mixed and the plate was incubated at 37°C for 2 h to allow the gels to polymerize. After polymerization, 0.5 mL of culture medium was added to each well to cover the gel, and the plate was incubated for an additional 48 h to enable gel contraction by the cells. The contraction index was quantified using ImageJ software (NIH, Bethesda, MD, USA).^27^

### 2.4 Transwell migration assays

A total of 1 × 10^5^ cells suspended in 150 μL of serum‐free medium were added to the Transwell inserts (Corning, Acton, MA, USA). Then, 750 μL of complete growth medium containing 10% FBS was added to the lower chamber to create a chemo‐gradient to attract cell migration. After 24 h of incubation, the cells were fixed with cold 100% methanol and stained with 0.1% crystal violet. The non‐migrated cells on the topside of the Transwell insert were gently removed using a cotton swab. The number of migrated cells on the underside was counted from five randomly selected fields under the microscope.^28^

### 2.5 RNA‐sequencing

Total RNA from three independent fBMFs, treated with or without carvacrol, was extracted using TRIzol™ Reagent following the manufacturer's protocol (Invitrogen Life Technologies, Carlsbad, CA, USA). The RNA quality for each sample was verified by Genomics Inc. After RNA‐seq library preparation and construction, changes in the transcriptome of the cells were analysed using the FPKM method (fragments per kilobase of transcript per million mapped reads) on the Illumina HiSeq platform (HiSeq2500, Illumina, San Diego, CA, USA) as previously described.^29^, ^30^

### 2.6 Real‐time quantitative polymerase chain reaction (qRT‐PCR)

Tissue and cell samples were processed for total RNA extraction using TRIzol™ Reagent following the protocol provided by the manufacturer (Invitrogen Life Technologies, Carlsbad, CA, USA). Subsequently, cDNA synthesis and quantitative polymerase chain reactions (qPCR) were carried out using the Superscript III First‐Strand Synthesis System (Invitrogen Life Technologies, Carlsbad, CA, USA) and the ABI StepOne™ Real‐Time PCR System (ThermoFisher Scientific, Carlsbad, CA, USA), respectively. The primer sequences utilized were as follows: PVT1, 5′‐CAAGCGAGGCCAGGTTTTTC‐3′ (forward) and 5′‐ CCACGAGGACACACATGGAA‐3′ (reverse); GAPDH, 5′‐CTCATGACCACAGTCCATGC‐3′ (forward) and 5′‐ TTCAGCTCTGGGATGACCTT‐3′ (reverse). Expression levels of PVT1 relative to GAPDH were determined using the delta Ct and comparative methods.

### 2.7 Western blot analysis

The cellular proteins were extracted by using 1xRIPA buffer supplemented with a protease and phosphatase inhibitor cocktail (Abcam, Cambridge, MA, UK). The protein concentration in each sample was quantified using the Bradford assay (Bio‐Rad Laboratories Inc., Hercules, CA, USA). For gel electrophoresis, 20 μg of protein from the cell lysates was loaded onto a 10% SDS‐polyacrylamide gel and transferred onto PVDF membranes (Millipore, Billerica, Massachusetts, USA). After blocking with 5% bovine serum albumin (BSA) at room temperature for 1 h, the membranes were incubated with primary antibodies overnight at 4°C, followed by secondary antibodies conjugated with HRP at room temperature for 1 h. The chemiluminescent signals of the immunoreactive bands were visualized using ECL chemiluminescent reagent and captured with a LAS‐1000plus Luminescent Image Analyzer (GE Healthcare Biosciences, Piscataway, NJ, USA). Primary antibodies utilized in this study include anti‐α‐SMA (Abcam, Cambridge, MA, USA), anti‐NLRP3 (Cell Signaling Technology, Danvers, MA, USA), anti‐cleaved‐GSDMD (Cell Signaling), anti‐cleaved‐IL‐1β (Cell Signaling), anti‐ASC (Cell Signaling) and anti‐GAPDH (GeneTex Inc., Irvine, CA, USA).

### 2.8 Lentiviral‐mediated silencing and overexpression of PVT1

To create the lentiviral vector for PVT1 silencing (Sh‐PVT1), oligonucleotide sequences targeting human PVT1 shRNA were synthesized and inserted into the pLV‐RNAi vector following the manufacturer's instructions (Biosettia, San Diego, CA, USA). The specific target sequences for PVT1 were Sh‐PVT1: 5′‐ AAAAGGATTCTTACAGCTTGGATTTGGATCCAAATCCAAGCTGTAAGAATCC ‐3′ and Sh‐PVT1‐2: 5′‐ AAAAGGCCTCGTGTCTATTAAATTTGGATCCAAATTTAATAGACACGAGGCC ‐3′. For the construction of the lentiviral vector for PVT1 overexpression (pLV‐PVT1‐cDNA), full‐length PVT1 cDNA was amplified using RT‐PCR and subsequently cloned into the pLV‐EF1a‐MCS‐IRES‐Puro vector (BioSettia). The pLV‐Sh‐PVT1 or pLV‐PVT1‐cDNA vectors were then co‐transfected with packaging and envelope vectors into 293 T cells using Lipofectamine 2000, as per the manufacturer's protocol (LF2000, Invitrogen, Carlsbad, CA, USA), to generate lentiviral particles. The overexpression and knockdown of PVT1 were performed by infecting cells with lentiviral particles containing either the full‐length PVT1 cDNA or the shRNA sequences targeting PVT1, respectively.^31^

### 2.9 Statistical analysis

Data were collected from a minimum of three independent experiments and are presented as mean ± standard deviation. Statistical significance of the differences was determined using Student's *t*‐test or analysis of variance (ANOVA) with the Statistical Package for the Social Sciences software (version 13.0, SPSS, Inc., Chicago, IL).

## 3 RESULTS

### 3.1 Carvacrol reduces the cell viability and lncRNA PVT1 expression of fBMFs

**FIGURE 1:**
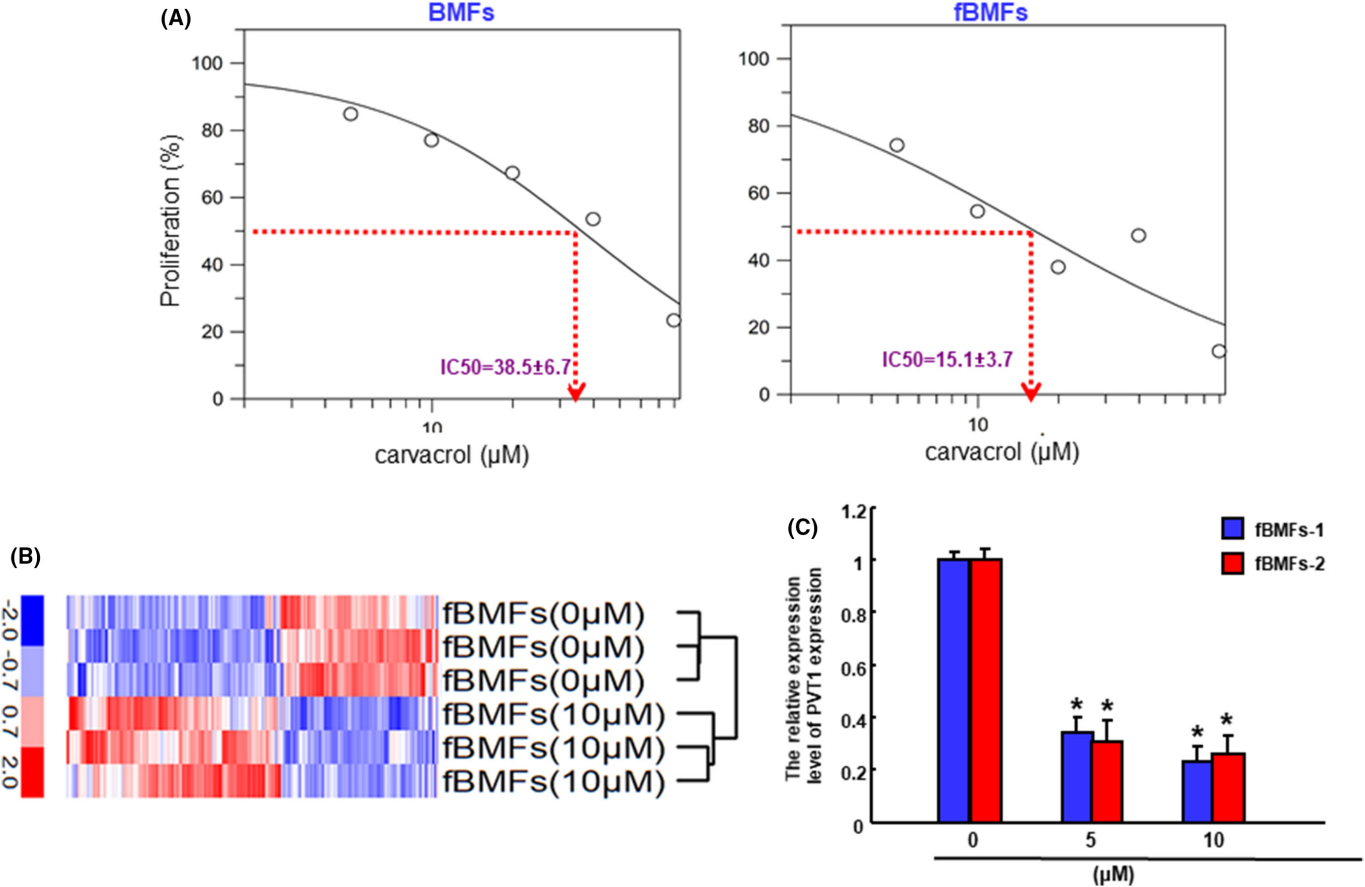
Carvacrol suppresses the cell proliferation rate and PVT1 expression of fBMFs. (A) The cytotoxicity effect of carvacrol in normal buccal mucosal fibroblasts (BMFs) and fibrotic BMFs (fBMFs). Cell survival/viability of BMFs (left panel) and fBMFs (right panel) were determined by MTT assay. IC50 values were calculated by GraFit software. (B) Hierarchical cluster heat map of the differentially expressed lncRNAs in RNA‐seq analysis of three paired fBMFs treated with or without 10 μM carvacrol. Red represented the upregulation of differentially expressed genes, and blue represented the downregulation of differentially expressed genes. (C) The relative expression of PVT1 in fBMFs treated with indicated concentrations of carvacrol. Results are means ± SD. *p < 0.05 compared to no treatment control group.

To determine the cytotoxic effect of carvacrol on normal BMFs and fBMFs‐derived from OSF tissues, the cell proliferation rate was assessed after treatment of various concentrations of carvacrol for 48 h using an MTT assay. Carvacrol exhibited a dose‐dependent suppressive property on cell survival in both BMFs and fBMFs, and the IC50 values for carvacrol in BMFs and fBMFs were 38.5 ± 6.7 and 15.1 ± 3.7 μM, respectively (Figure 1A). This result suggested that a lower concentration of carvacrol was sufficient to inhibit the cell survival of fBMFs without inducing severe cell toxicity of normal oral cells. Also, carvacrol was used in the following experiments within this concentration range to examine if it holds anti‐fibrotic potential. As shown in Figure 1B, a hierarchical cluster heat map of the differentially expressed lncRNAs in RNA‐seq analysis of three paired fBMFs treated with or without 10 μM carvacrol was presented, and PVT1 was one of the differentially expressed lncRNAs. Moreover, we measured the expression of PVT1 in fBMFs treated with 5 and 10 μM carvacrol, and found that PVT1 was markedly repressed in the carvacrol‐incubated fBMFs (Figure 1C).

### 3.2 Carvacrol mitigates the myofibroblast activation and pyroptosis of fBMFs

**FIGURE 2:**
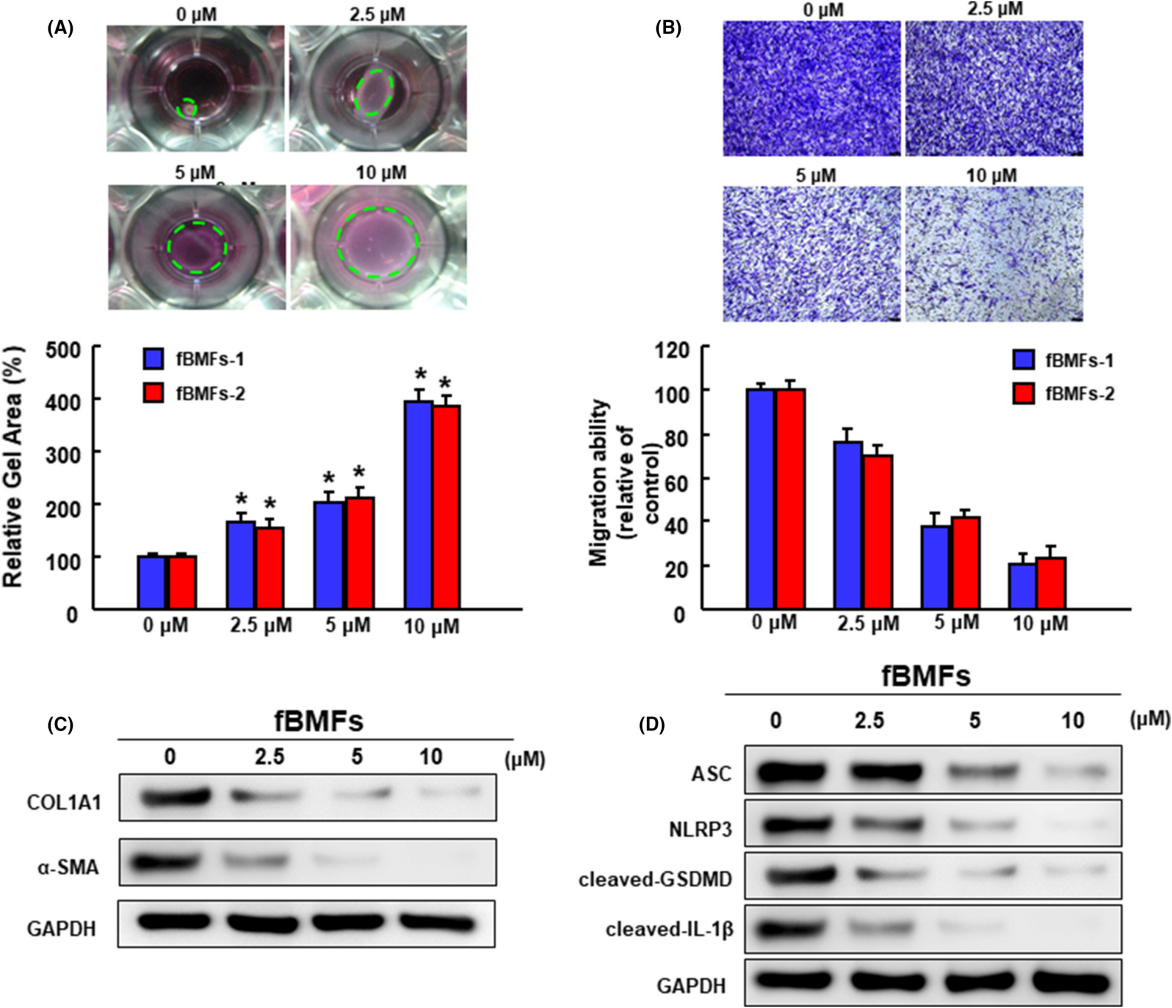
Carvacrol treatment decreases the myofibroblast activities and expression levels of fibrosis and pyroptosis of fBMFs. (A) The contractile capacity of fBMFs was determined by collagen gel contraction assay (three replicates for each concentration). Images of gels were captured and gel areas (dotted circles) were calculated by ImageJ software. (B) fBMFs were treated with the indicated concentration of carvacrol followed by transwell migration assay. The experiments were repeated for three times and data from a representative experiment were presented. Results are means ± SD. *p < 0.05 compared to control group. The expression of firosis (C) and pyroptosis (D) markers were evaluated by Western blot.

Following injury, the activated myofibroblasts will proliferate and migrate into the wound site to reconstitute the ECM components and close the wound. Accordingly, we examined the effects of carvacrol on collagen gel contractility, a well‐established assay to examine the fibroblast‐matrix interactions by Bell et al.,^32^ of fBMFs. We observed that the relative gel area was increased in fBMFs treated with carvacrol in a concentration‐dependent fashion, indicating that a higher dose of carvacrol attenuated the contractile activity of fBMFs (Figure 2A). Besides, fBMFs were subjected to transwell migration assay, and carvacrol dose‐dependently mitigated the migration capacity of fBMFs (Figure 2B). Aside from myofibroblast phenotypes, we also assessed the expression of fibrosis markers and showed that the expression levels of alpha‐1 type I collagen (COL1A1; the main type of collagen in OSF lesions^33^) and alpha‐smooth muscle actin (α‐SMA; the myofibroblast marker^34^) were gradually abrogated by carvacrol (Figure 2C). Moreover, the expression levels of pyroptosis markers, including ASC, NLRP3, cleaved‐GSDMD and cleaved‐IL‐1β, were dose‐dependently attenuated in the carvacrol‐treated fBMFs (Figure 2D).

### 3.3 PVT1 is aberrantly upregulated in OSF and the silencing of PVT1 diminishes myofibroblast activation and the expression of pyroptosis markers

**FIGURE 3:**
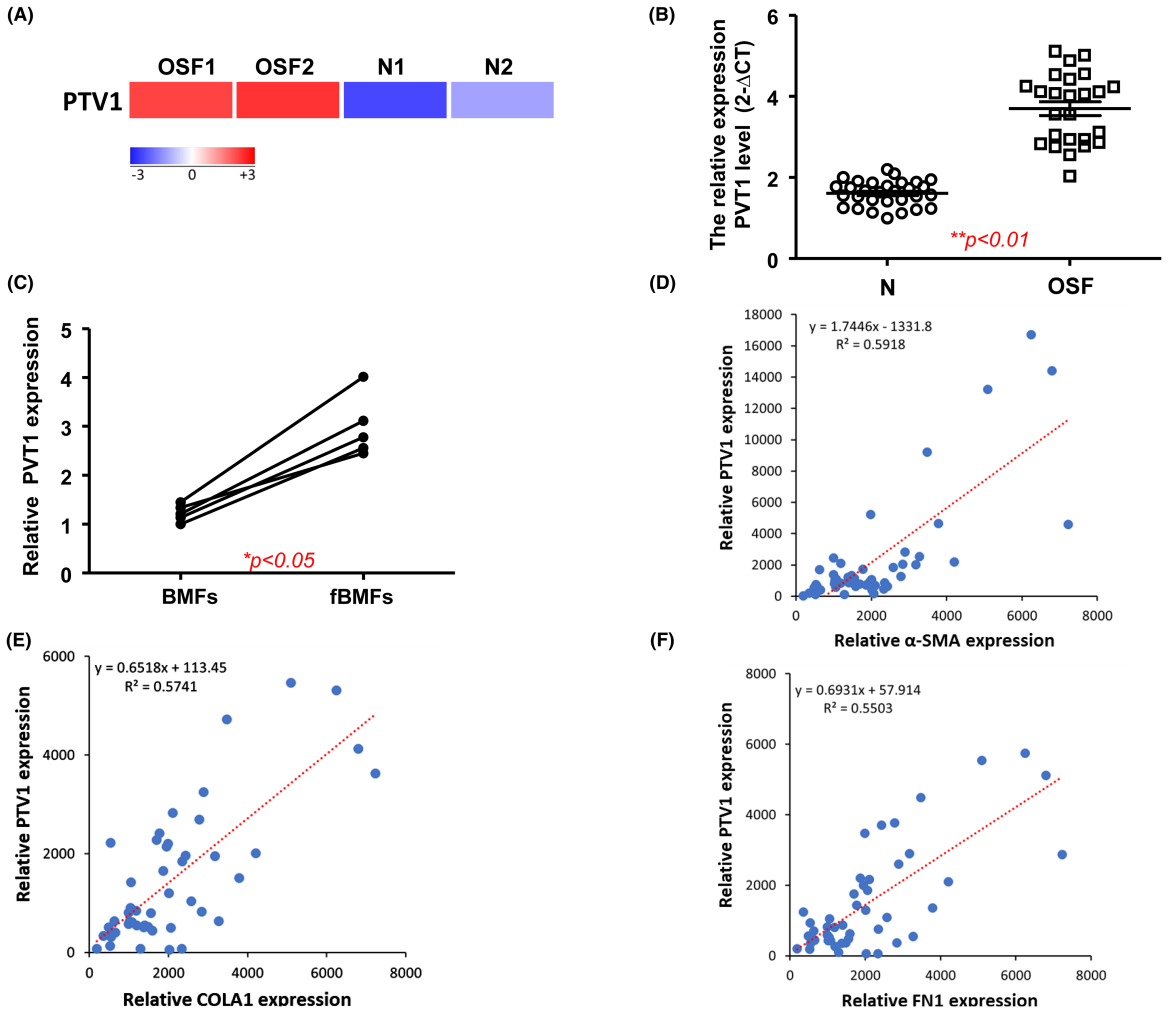
PVT1 is upregulated in OSF specimens and positively correlated with various fibrosis markers. (A) A heatmap showing PVT1 was highly expressed in two OSF tissues. (B) Gene expression of PVT1 in OSF samples and normal buccal mucosal tissues (*n* = 25). (C) Gene expression of PVT1 in BMFs and fibrotic BMFs derived from OSF tissues. *p < 0.05 compared to control. **p < 0.01. PVT11 was positively associated with numerous fibrosis‐related markers, including alpha smooth muscle actin (α‐SMA; D), type I collagen alpha 1 (COL1A1; E), and fibronectin (FN1; F).

By using RNA‐seq analysis, we noticed that lncRNA PVT1 was aberrantly overexpressed in the OSF specimens compared to normal mucosa (Figure 3A). We then verified our finding using qRT‐PCR and demonstrated that PVT1 was elevated in OSF samples (Figure 3B) and fBMFs derived from OSF tissues (Figure 3C). Furthermore, we found that the expression of PVT1 was positively correlated with various fibrosis markers, including α‐SMA (Figure 3D), COL1A1 (Figure 3E) and fibronectin (FN) (Figure 3F). These findings indicated that PVT1 may exert a pro‐fibrosis property in the development of OSF.

**FIGURE 4:**
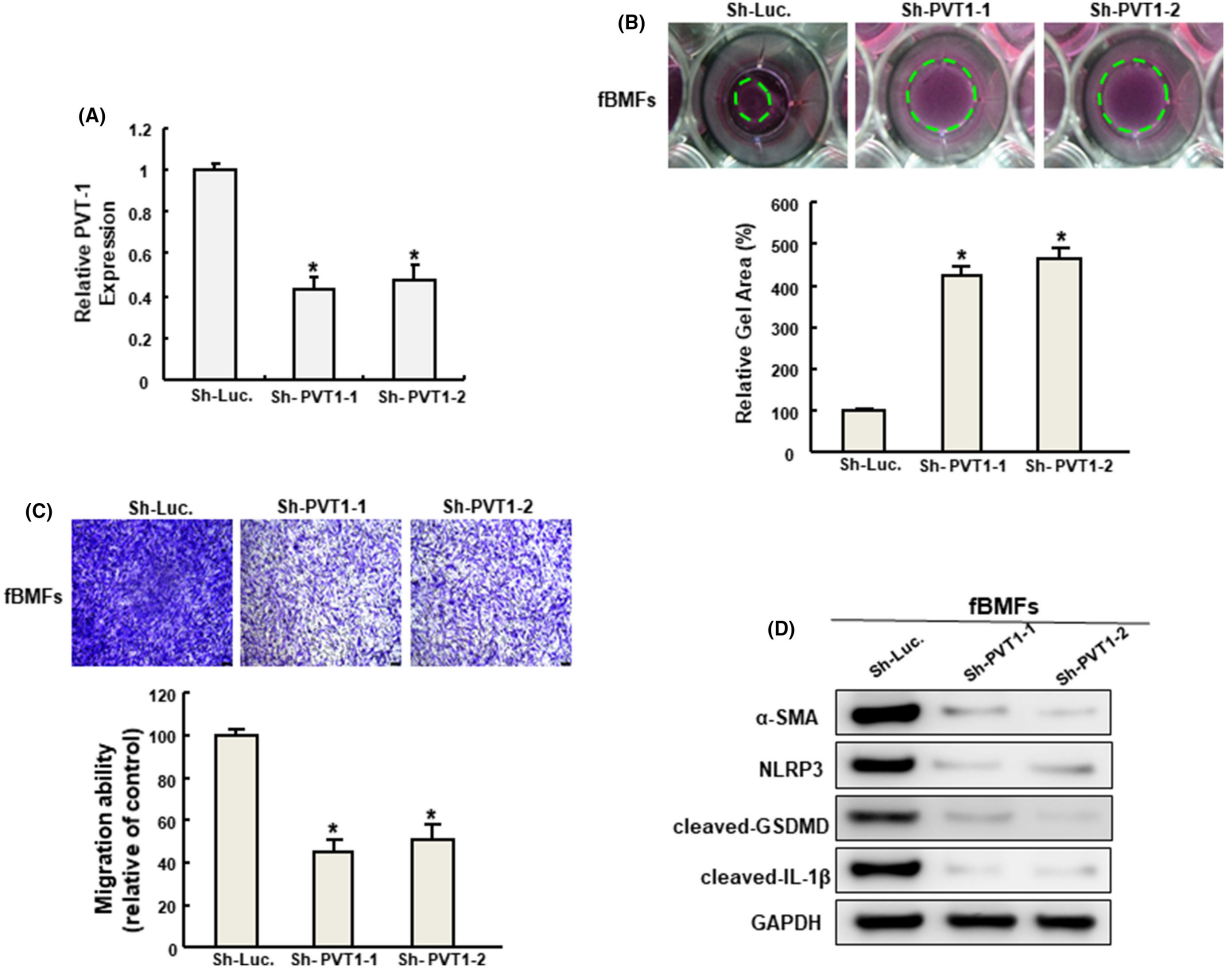
Silencing of PVT1 downregulates myofibroblast features and the expression of myo‐fibroblast and pyroptosis markers. Inhibition of PVT1 in fBMFs (A) diminished the myofibroblast activities, such as collagen gel contraction (B) and transwell migration (C). The protein expression of α‐SMA, NLRP3, cleaved‐GSDMD and cleaved‐IL‐1β in fBMFs silence for PVT1 (D).

In an attempt to verify our postulation, we examined the functional role of PVT1 on various myofibroblast features and pyroptosis markers. We showed that the silencing of PVT1 (Figure 4A) markedly relieved the collagen gel contraction (Figure 4B) and transwell migration (Figure 4C) capacities of fBMFs. Additionally, we found that the expression of myofibroblast marker, α‐SMA, was downregulated in cells silenced for PVT1 (Figure 4D). Furthermore, multiple pyroptosis markers, including NLRP3, cleaved‐GSDMD and cleaved‐IL‐1β were all abolished in fBMFs when PVT1 was silenced (Figure 4D).

### 3.4 The suppressive property of carvacrol on myofibroblast activities and pyroptosis is mediated by PVT1

**FIGURE 5:**
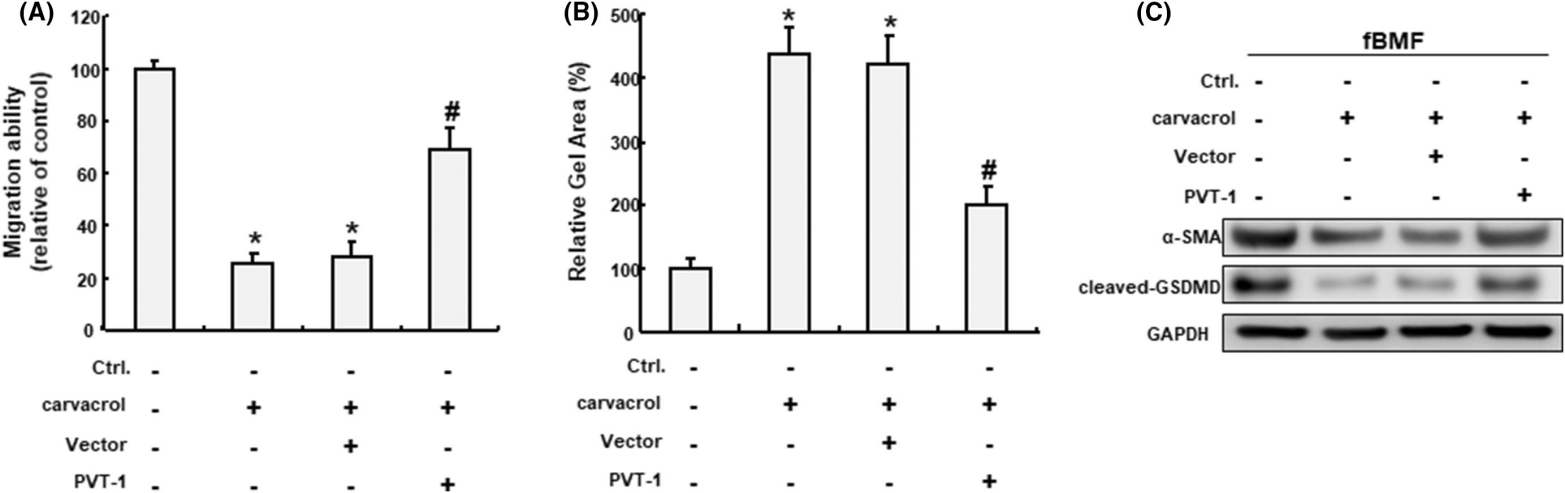
Ectopic expression of PVT1 reverses the effects of carvacrol on myofibroblast activation and cleaved‐GSDMD expression. Administration of carvacrol suppressed the transwell migration (A), collagen gel contraction (B) and protein expression of α‐SMA and cleaved‐GSDMD (C), whereas forced expression of PVT1 abolished these effects. *p < 0.05 compared to control. #*p* < 0.05 compared to carvacrol‐only group.

Given that PVT1 was downregulated in the carvacrol‐treated fBMFs and suppression of PVT1 resulted in a decrease in myofibroblast activation and pyroptosis, we investigate whether the inhibitory effect of carvacrol on the myofibroblast activation and pyroptosis of fBMFs was mediated by modulation of PVT1. In the rescue experiments, we showed that the lower migration ability of fBMFs in response to carvacrol was reversed when cells were overexpressed for PVT1 (Figure 5A). Besides, the reduced collagen gel contractility in the carvacrol‐treated fBMFs was mitigated when PVT1 was forced expressed (Figure 5B). Likewise, carvacrol treatment inhibited the expression of α‐SMA and cleaved‐GSDMD in fBMFs, while overexpression of PVT1 countetacted this effect (Figure 5C). Collectively, these results demonstrated that PVT1 mediated the carvacrol‐induced repression on myofibroblast activation and pyroptosis.

### 3.5 PVT1 modulates myofibroblast activation and the expression of pyroptosis markers by sponging miR‐20a

**FIGURE 6:**
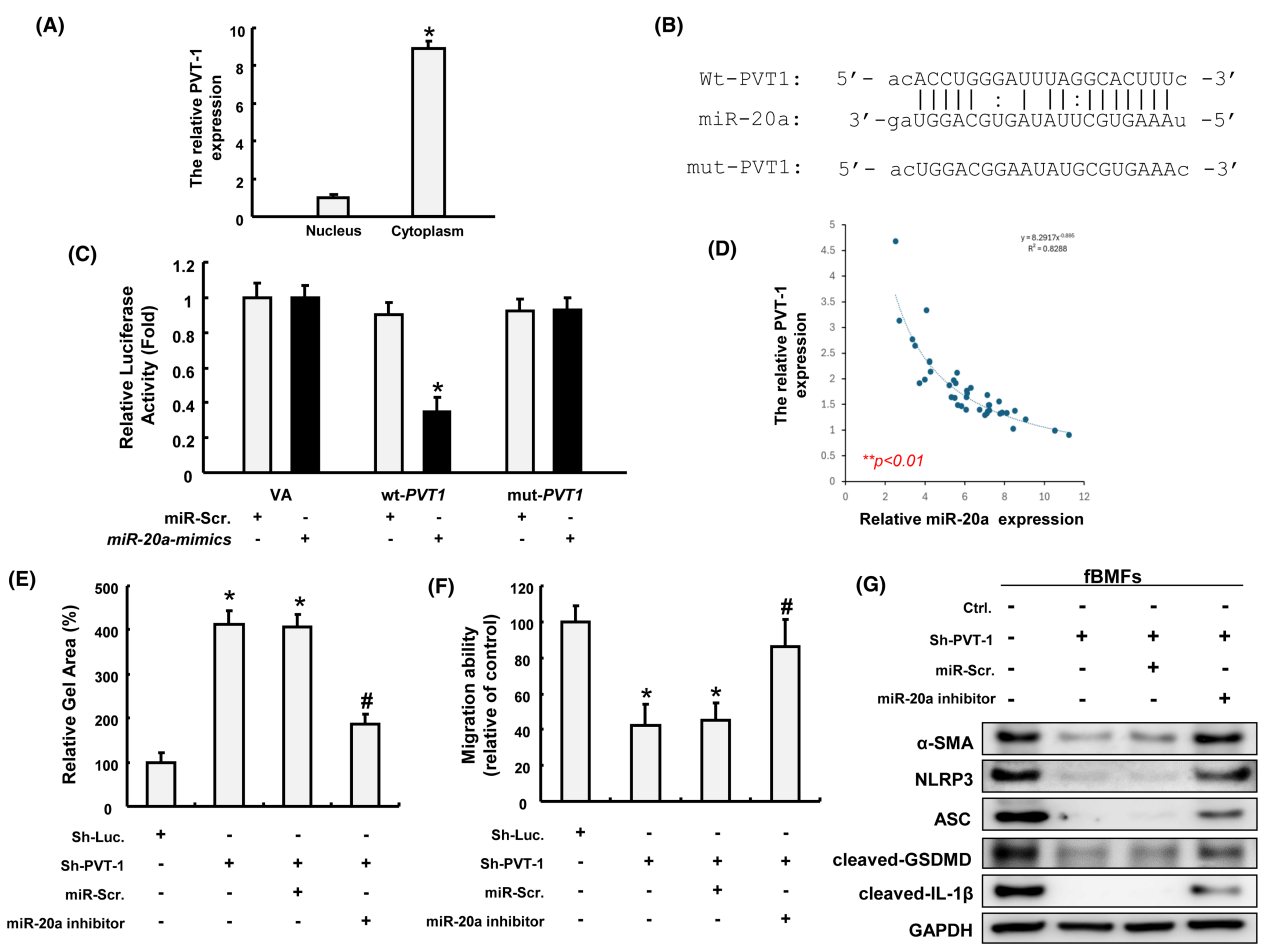
PVT1 regulates fBMFs activation and pyroptosis by direct interaction with miR‐20a. (A) The subcellular localization of PVT1 in fBMFs analysed by qRT‐PCR. *p < 0.05 compared to the nucleus group. (B) Schematic representation of the alignment of the PVT1 base pairing with miR‐20a. Wild‐type (Wt) and mutated (Mut) PVT1 reporter plasmids were co‐transfected with miR‐20a or empty vectors (miR‐Scr.). (C) The relative luciferase activity of each combination in two fBMFs is assessed and the activity was only reduced in fBMFs co‐transfected with wt‐PVT1 and miR‐20a. (D) A negative correlation between PVT1 and miR‐20a was observed. Inhibition of miR‐20a in fBMFs reverted the myofibroblast activities, including collagen gel contraction (E) and migration ability (F) in fBMFs following the silencing of PVT1. (G) The expression of myofibro‐blast marker (e.g. α‐SMA) and pyroptosis markers (e.g. NLRP3, ASC, cleaved‐GSDMD and cleaved‐IL‐1β) were all suppressed in fBMFs with knockdown of PVT1, which was prevented in the presence of miR‐20a inhibitor.

After validating PVT1 involved in the carvacrol‐mediated suppression of myofibroblast activities and pyroptosis, we then investigated how PVT1 exerted these effects. First, we analysed the extracted RNAs from the cytoplasmic and nuclear fractions of fBMFs using qRT‐PCR, and our results showed that PVT1 was predominantly distributed in the cytoplasm of fBMFs (Figure 6A). One of the recent studies has demonstrated that PVT1 modulates pyroptosis in septic acute kidney injury by targeting miR‐20a‐5p.^16^ Herein, a luciferase reporter assay was conducted to ensure that PVT1 directly competed with miR‐20a. As shown in Figure 6B, the binding sites between PVT1 and miR‐20a were predicted and mutated (mut) PVT1 3′UTR was constructed as well. Compared with the miR‐Scr. group, lower luciferase activity was detected from cells co‐transfected with the wild‐type (wt)‐PVT1 vector and miR‐20a mimics, whereas no obvious change was displayed in cells with the mut‐PVT1 vector (Figure 6C). In agreement with this finding, we observed that there was a negative relationship between PVT1 and miR‐20a (Figure 6D). These results suggested that PVT1 may act as a ceRNA of miR‐20a.

To address whether miR‐20a mediated the modulation of PVT1 on myofibroblast activation and pyroptosis, miR‐20a inhibitor was employed to examine the effect of knockdown of PVT1. As expected, silencing of PVT1 decreased collagen gel contractility, while the reverse phenomenon was observed when miR‐20a inhibitors were introduced into fBMFs (Figure 6E). Similarly, fBMFs with sh‐PVT1 exhibited a significant reduction of cell migration capacity, while co‐transfection of miR‐20a inhibitor reversed it (Figure 6F). In addition, we found that the expression of α‐SMA, NLRP3, ASC, cleaved‐GSDMD and cleaved‐IL‐1β were all suppressed in fBMFs silenced for PVT1 (Figure 6G). However, these effects were blocked when cells were co‐transfected with miR‐20a inhibitor (Figure 6G), which was in support of the above interpretation that PVT1 acts as a molecular sponge for miR‐20a to regulate myofibroblast activation and pyroptosis.

## 4 DISCUSSION

**FIGURE 7:**
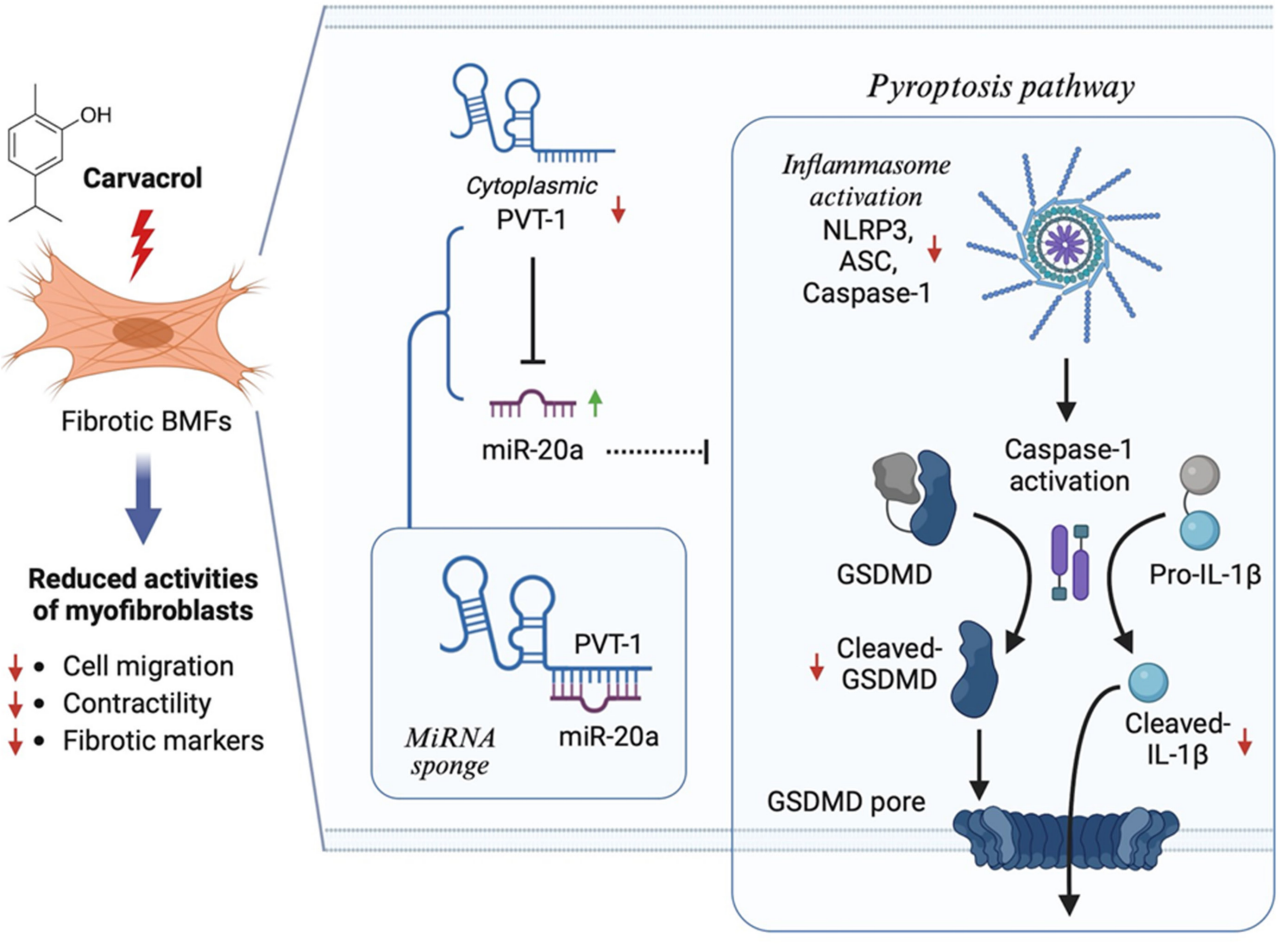
Schematic representation of the therapeutic potential of carvacrol in OSF. Carvacrol may lower pyroptosis and the susequent myofibroblast activation via targeting the PVT1/ miR‐20a axis, leading to downregulation of NLRP3, ASC, cleaved‐GSDMD and cleaved‐IL‐1β as well as myofibroblast activities.

Carvacrol (5‐isopropyl‐2‐methylphenol) is a monoterpenic phenol primarily found in essential oils of oregano (Origanum vulgare), thyme (Thymus vulgaris) and other plants with various pharmacological properties, such as anti‐cancer, anti‐microbial and anti‐oxidant effects.^35^ Numerous studies have revealed that carvacrol displayed suppressive effects on oral cancer cells.^24^, ^36^ In addition, lots of studies have shown that carvacrol exhibited anti‐fibrosis properties in liver,^22^, ^37^ kidney^38^ and cardiac^39^ fibrosis. Carvacrol has been found to reduce the proliferation and activation of hepatic stellate cells induced by PDGF‐BB^37^ and inhibit the expression of α‐SMA in the kidney of unilateral ureteral obstruction mouse model.^38^ One of the recent studies also showed that carvacrol protects the heart against sepsis‐induced myocardial dysfunction by suppressing pyroptosis via NLRP3/caspase1/GSDMD signalling.^40^ Moreover, several studies have demonstrated that the administration of carvacrol diminished the NLRP3 inflammasome activation.^36^, ^41^, ^42^ In agreement with these findings, our data suggested that carvacrol treatment may be a feasible approach to hinder the progression of precancerous OSF as the administration of carvacrol successfully attenuated the expression of markers of NLRP3 inflammasome‐mediated pyroptosis, including NLRP3, ASC, cleaved GSDMD and cleaved IL‐1β. Also, we demonstrated that the myofibroblast phenotypes and the expression of fibrosis markers in fBMFs were all downregulated in the presence of carvacrol. Most importantly, our data indicated that the inhibitory effects of carvacrol on pyroptosis and myofibroblast characteristics were mediated by regulation of the PVT1/miR‐20a axis (Figure 7).

LncRNA PVT1 was first discovered as a breakpoint site in variant (6;15) translocations, which is associated with murine plasmacytomas.^43^ The human PVT1 gene begins 57 kilobase pairs downstream of the *MYC* oncogene and resides in the chromosomal locus 8q24.21.^44^ PVT1 encodes a series of annotated non‐coding RNAs, including 26 linear and 26 circular isoforms, and 6 miRs,^45^ and acts as an epigenetic modulator, transcription, posttranscriptional regulators, enhancer of mRNA and protein stability and miR sponges.^46^ Several studies have revealed that PVT1 promoted the progression of oral cancer by serving as a ceRNA to target the miR‐375/YAP1^21^ or miR‐150‐5p/GLUT‐1^47^ axes. Besides, PVT1 has often been regarded as a fibrosis factor as it was predominantly upregulated in various types of fibrosis diseases, such as cardiac^48^, ^49^ and renal^50^ fibrosis. It has been shown that the expression of PVT1 in myofibroblasts appeared to be pivotal in terms of cell proliferation, migration, activation and expression of fibrosis markers. For instance, it has been demonstrated that PVT1 promoted the transforming growth factor‐β1 (TGF‐β1)‐β1‐induced proliferation and activation of human cardiac fibroblasts^48^ and lung fibroblasts.^51^ PVT1 also mediated the TGF‐β1‐induced upregulation of α‐SMA and fibronectin in proximal tubular HK‐2 cells^50^ and angiotensin II‐elicited collagen production along with the TGF‐β1/Smad signalling activation in atrial fibroblasts.^49^ Apart from regulating myofibroblast activation, PVT1 participated in pyroptosis in vascular smooth muscle cells,^52^ myoblast H9C2 cells following I/R damage^53^ and lipopolysaccharide (LPS)‐induced HK‐2 cells^16^ as well. Our findings were consistent with these results, showing that PVT1 was aberrantly overexpressed in OSF specimens or fBMFs derived from these fibrosis tissues. We not only demonstrated that downregulation of PVT1 resulted in lower myofibroblast features and expression of pyroptosis markers, but also revealed that PVT1 exerted its fibrosis activity by directly binding and titrating miR‐20a. Moreover, It has been reported that the prognosis of patients with head and neck squamous cell carcinoma showing high PVT1 expression was poor,^21^ and we also found that PVT1 was upregulated in OSF specimens. The clinical implications of using PVT1 as a diagnostic or prognostic marker for malignant transformation of precancerous OSF is worthy of additional research.

Various studies have uncovered that miR‐20a plays a regulatory role in the development of several cancers.^54^ For example, the upregulated miR‐20a‐5p expression in head and neck squamous cell carcinoma cells was proven to elicit cell proliferation, migration and invasion through the death receptor 6/ C‐C motif chemokine receptor 7 axis.^55^ Nevertheless, opposite findings were reported that miR‐20a was one of the tumour‐suppressive miRNAs identified in high‐grade gliomas, and decreased expression of miR‐20a was associated with a better survival probability for both primary and recurrent glioma patients.^56^ Besides, miR‐20a was found to inhibit the aggressiveness of oral cancer cells, and there was a negative correlation between miR‐20a and TNM stage as well as lymphatic metastasis.^57^ As for fibrosis, the majority of the studies suggested that miR‐20a exhibited an inhibitory feature. For instance, miR‐20a‐5p mimic has been shown to attenuate I/R injury and postischemic renal fibrosis by negatively regulating the acyl‐CoA synthetase long‐chain family member 4.^58^ The miR‐20a‐5p level was markedly downregulated in hepatic stellate cells after TGF‐β1 treatment or liver fibrosis samples, and elevation of miR‐20a‐5p reduced liver fibrosis by directly targeting TGFBR2^59^, ^60^ or phosphatase and tensin homologue (PTEN).^61^ Downregulation of miR‐20a‐p was also discovered in the pulmonary tissues of ovalbumin‐treated mice, and overexpression of miR‐20a‐5p in the ovalbumin‐treated cells mitigated fibrosis and inflammatory response by directly targeting ATG7.^62^ Furthermore, accumulating research also suggested that miR‐20a was implicated in the regulation of pyroptosis. It has been shown that transfection of miR‐20a mimic reversed the high glucose‐induced pyroptosis and inflammatory factors in retinal pigment epithelium cells by directly inhibiting TXNIP.^63^ In addition, the expression of miR‐20a‐5p was markedly reduced in sepsis model mice and LPS‐induced HK‐2 cells. Deng et al. showed that knockdown of miR‐20a‐5p aggravated the LPS‐induced cell pyroptosis by directly targeting NLRP3.^16^ Collectively, these results were in support of our findings that the presence of miR‐20a was essential for the suppressive effects of silencingof PVT1 on myofibroblast activation and pyroptosis. It is plausible to assume that miR‐20a may directly bind to NLRP3 to decrease its expression in our case, but further investigation was required to verify this presumption.

Of note, several limitations exist in using cell lines to investigate the effects of carvacrol on the progression of OSF. For instance, it lacks the complex architecture and microenvironment found in fibrosis tissues, as well as limits the interaction with other cell types through various signalling pathways and extracellular matrix components. Also, cell lines that are derived from a single individual may result in a deficiency of genetic diversity and make them less representative of OSF in its native context. Given that cell lines typically fail to capture the interactions and contributions of other cell types or systemic conditions involved in OSF, it is necessary to conduct an animal study to confirm our findings in the future.

Taken together, we showed that PVT1 was aberrantly upregulated in the OSF specimens and may contribute to oral fibrogenesis. Our results suggested that PVT1 was one of the repressed non‐coding RNAs in fBMFs treated with carvacrol, and the subsequent experiments demonstrated that carvacrol may be a good candidate for therapeutic applications in OSF by regulation of pyroptosis and myofibroblast activation through the PVT1/ miR‐20a axis.

## AUTHOR CONTRIBUTIONS

**Cheng‐Chia Yu:** Conceptualization (equal); writing – review and editing (equal). **Pei‐Ling Hsieh:** Formal analysis (equal); methodology (equal); writing – review and editing (equal). **Shih‐Chi Chao:** Data curation (equal); investigation (equal). **Yi‐Wen Liao:** Data curation (equal); formal analysis (equal); methodology (equal). **Chuan‐Hang Yu:** Formal analysis (equal); validation (equal); visualization (equal). **Pin Ju Chueh:** Supervision (equal). **Chih‐Yu Peng:** Funding acquisition (equal); supervision (equal). **Shiuan‐Shinn Lee:** Conceptualization (lead); supervision (equal).

## CONFLICT OF INTEREST STATEMENT

The authors declare that they have no conflict of interests.

## Data Availability

The data that support the findings of this study are available from the corresponding author upon reasonable request.
